# A Novel Personalized Strategy for Hip Joint Flexion Assistance Based on Human Physiological State

**DOI:** 10.3390/bios14090418

**Published:** 2024-08-27

**Authors:** Beixian Wu, Chunjie Chen, Sheng Wang, Hui Chen, Zhuo Wang, Yao Liu, Tingwei He, Jiale Zhang, Xinyu Wu

**Affiliations:** 1 College of Mechanical and Control Engineering, Guilin University of Technology, Guilin 541006, China; bx.wu3@siat.ac.cn (B.W.); zjlzjl291@126.com (J.Z.); 2 ShenZhen Institute of Advanced Technology, Chinese Academy of Sciences, Shenzhen 518055, China; s.wang3@siat.ac.cn (S.W.); h.chen2@siat.ac.cn (H.C.); zhuo.wang@siat.ac.cn (Z.W.); ll.liu1@siat.ac.cn (Y.L.); tw.he@siat.ac.cn (T.H.); xy.wu@siat.ac.cn (X.W.); 3 Guangdong Provincial Key Lab of Robotics and Intelligent System, Shenzhen Institute of Advanced Technology, Chinese Academy of Sciences, Shenzhen 518055, China; 4 Guangdong-Hong Kong-Macao Joint Laboratory of Human-Machine Intelligence-Synergy Systems, Shenzhen 518055, China

**Keywords:** soft exosuit, wearable sensor monitoring, metabolic cost, hip flexion, status of physical signs

## Abstract

Soft exosuits have emerged as potent assistive tools for walking support and rehabilitation training. However, most existing soft exosuit systems rely on preset assistance modes, which may not accurately align with individual physiological states and movement requirements, leading to variable user experiences and efficacy. While existing human-in-the-loop (HIL) research predominantly focuses on optimizing metabolic cost and torque difference parameters, there is a notable absence of real-time monitoring methods that closely reflect the human body’s physiological state and strategies that dynamically indicate walking efficiency. Motivated by this, we developed a novel personalized power-assist system. This system optimizes the power-assist output of the hip joint by monitoring the user’s physiological and motion signals in real time, including heart rate (HR), blood oxygen saturation (SpO_2_), and inertial measurement unit (IMU) data, to assist hip flexion based on feedback. The findings from a metabolic expenditure trial demonstrate that the innovative soft exosuit, which is based on a Physiological State Monitoring Control (PSMC) system, achieves a reduction of 7.81% in metabolic expenditure during treadmill walking at a speed of 3.5 km/h compared to walking without the assistance of the exosuit. Additionally, during continuous exercise with varying intensities, the metabolic consumption level is reduced by 5.1%, 5.8%, and 8.2% at speeds of 2, 4, and 6 km per hour, respectively. These results support the design of a novel hip flexion-assisting soft exosuit, demonstrating that applying different assistance forces in consideration of different physiological states is a reasonable approach to reducing metabolic consumption.

## 1. Introduction

Throughout history, humanity has harnessed tools to augment physical capabilities and mobility, which are crucial for self-improvement and global exploration. Over the past few decades, wearable robots and health monitoring devices have gained popularity due to their potential to aid human locomotion in rehabilitative and occupational settings [1,2,3]. The decline in physical function due to aging, the alteration of gait patterns, and the subsequent sedentary lifestyle are common issues. Particularly, post-COVID-19 cardiopulmonary illnesses have exacerbated this decline, increasing the risk of cardiovascular and metabolic disorders, thereby diminishing mobility and potentially leading to secondary health issues such as obesity and depression [4,5]. Wearable robotic assistive devices offer a solution by supporting gait, thereby alleviating the load on the neuromuscular system. While breakthroughs in actuation, computation, and machine learning have accelerated the development of wearable devices, the challenge of customizing control strategies for seamless human–machine interaction remains [6]. This has limited the integration of wearable robots in everyday use. Enhancing bidirectional interactive performance between devices and users is key to maximizing the potential of wearable robot technology [7]. In 2018, research introduced a comprehensive wearable health monitoring solution that integrates a glucose-sensing strip designed for sweat analysis and a sophisticated smart bracelet. The sweat-based glucose strip is affixed to the forehead to monitor glucose levels, while the smart bracelet, equipped with advanced electronic components, is worn on the wrist to track vital signs, including blood oxygen levels, pulse rates, and physical exertion [8,9]. Beyond the conventional electrical sensors for physiological data collection, the system also employs acoustic sensing technology utilizing ultrasonic transducers to capture a broader range of intricate hemodynamic indicators [10]. Historically, user preferences have played an informal role in the evolution of assistive technology, but researchers are now formally incorporating these preferences into system design and adjustment processes. The integration of human–machine interaction based on user preferences allows for a dynamic approach in which users can customize device assistance according to a holistic view of physiological, kinematic, and biomechanical objectives, thereby tailoring the device to their personal needs. This has shown significance in enhancing device utility and user acceptance [6].

Exoskeletons, highly reliant on the user’s movement patterns and physiological state, enhance physical capabilities by providing additional force parallel to muscle [11,12,13]. Hao Su and colleagues developed a novel approach that eliminates the need for experiments to cultivate a flexible control policy within a simulated environment. This innovative method effectively narrows the divide between simulated scenarios and real-world applications, all without the necessity for human-based experiments [14]. Optimizing their interactive performance is vital for improving user experience, ensuring safety, and increasing locomotive efficiency. Designing stiff and flexible exoskeletons that target certain joints or activities requires understanding human interaction with wearable technologies and sports physiology. Most lower-limb exoskeletons feature rigid structures that provide significant assistance to patients with limited mobility, but they can restrict degrees of freedom and affect wearer comfort. In contrast, soft exosuits are favored by researchers for their flexibility. For instance, a hip exoskeleton controller developed by Enrica Tricomi and colleagues can distinguish different walking terrains using an RGB camera and adjust assistance adaptively [15]. Soft HIL exosuits utilize real-time measured physiological signals, such as emotion, to continuously optimize control parameters and generate customized assistance patterns [16]. Ding Ye and colleagues evaluated a reconfigurable multi-joint exosuit through sports biomechanics and physiology, minimizing the exoskeleton’s alteration of the wearer’s natural movement, reducing electromyography (EMG) activity in most muscles, and achieving higher metabolic reduction under multi-joint assistance conditions [17]. While these studies adjust assistance patterns by sensing environmental or physiological information, none have highlighted the use of gait parameters and physiological states as feedback mechanisms for exoskeleton control to achieve personalized assistance. Therefore, this work offers a personalized lower-limb soft exosuit system that takes into account human physiological conditions and gait factors to improve human–machine interaction performance.

Heart rate (HR) and blood oxygen saturation (SpO_2_) serve as critical biomarkers for assessing an individual’s physiological condition, playing significant roles in the realms of sports medicine and health monitoring. Studies have demonstrated that these biomarkers are instrumental in enhancing the body-in-the-loop functionality of exoskeleton robotics. Previous research developed an optimization algorithm based on real-time estimation of energy expenditure through indirect calorimetry, which could automatically fine-tune the parameters for optimal ankle assistance during walking. However, this method is not suited for long-term use and is confined to laboratory settings [18]. Subsequently, a systematic evaluation method was proposed, utilizing a variety of portable physiological sensors, including HR and SpO_2_, to predict energy expenditure, demonstrating promising predictive performance. Yet, this method has not been applied in exoskeleton experiments for human–machine interaction [19]. Moreover, existing studies have indicated a correlation between changes in gait performance and both physiological and psychophysical measurements. This suggests that perceived fatigue at higher walking speeds, reflected by HR response and localized muscular discomfort, may lead to an earlier perceptual crossover due to increased metabolic demands.

Research shows that increasing walking speed improves both objective and subjective gait performance indicators, including hip and knee joint movements. Peak sagittal plane kinematic parameters (extension and flexion) have a significant correlation with gait velocity [17]. It has been observed that brisk walking increases the impact of heel strikes, imposing additional dynamic load on the musculoskeletal system and potentially enhancing the perception of whole-body movement. This research supports the theory that gait dynamics are crucial for developing ’on-demand’ exoskeleton assistance systems that provide torque only when there is a significant deviation from the required movement. Despite advancements, the application of combining physiological signals with gait parameters for real-time optimization remains largely unexplored. To the best of our knowledge, no exoskeleton studies have considered personalized assistance based on human physiological states under varying exercise intensities. Therefore, this study introduces HR, SpO_2_, and gait parameter characteristics as a real-time feedback mechanism in soft exosuit human–machine interaction. The soft exosuit system we propose not only provides assistance based on motion data but also intelligently adjusts according to the user’s real-time physiological state, achieving more personalized and efficient assistance. This approach is expected to enhance user performance, reduce exercise-related risks, and improve the overall exercise experience.

The primary contributions of this study are centered around a key challenge in the field of wearable robotics: enhancing the adaptability and responsiveness of soft exosuit robots to individual physiological states. Our work introduces the following advancements: (1) the development of a soft exosuit robot capable of integrating gait parameters with real-time physiological data to enhance the synergy between humans and robots, and (2) a novel control algorithm that allows for real-time adjustments to the exosuit’s assistance based on dynamic physiological changes in various walking environments. Section 2 outlines a systematic approach to exosuits. Then, Section 3 addresses the aid strategy and control algorithm. In Section 4, we present the experimental and analytical results. Our novel soft exosuit is discussed in Section 5. Finally, Section 6 presents the conclusions.

## 2. Exosuit Hardware Setup

**Figure 1:**
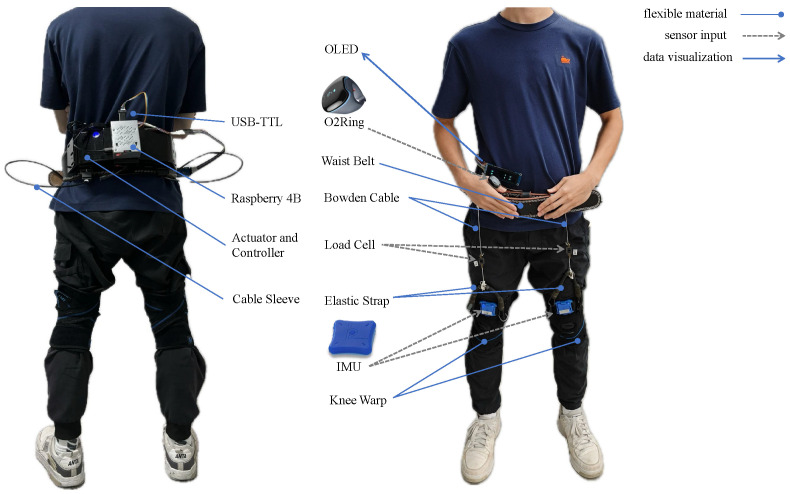
The design and prototype of the proposed soft exosuit.

As shown in Figure 1, the soft exosuit designed by us feeds the real-time signal back into the control loop online, which addresses the problem of the open loop caused by the poor feedback effect of tension sensors in traditional systems. The main purpose is to accommodate healthy adults’ exercise preferences based on their physical conditions in different motion states. Wearers can benefit from rehabilitation therapy and reduced metabolic expenditure.

The system is appropriate for healthy individuals, including teenagers and the elderly, and places fewer limits on the wearer because it is primarily composed of flexible textile materials. The experimental study utilized a 2.95 kg lightweight exosuit for the lower limb that was specifically developed to assist in hip flexion. The textile components of the covering consist of a waistband and two leg sections fastened to the knee joint. The central control unit, actuators, and power source are positioned at the rear of the waistband, while the anterior section of the waistband features proximal attachment points created using 3D printing. Furthermore, the front portion of the leg harness is connected to the distal attachment point. Table 1 shows the locations and masses of the primary components.

### 2.1. Design of The Proposed Soft Exosuit

The IMU (LPMS-B2, LPRESEARCH, Guangzhou, China) contains a gyroscope and an accelerometer, which are the key sensors for collecting human gait information [20]. The thigh IMU module is located on the front side of the brace. By capturing and acquiring the gait cycle, the gait intersection angle is directly fed back to the control system as a signal. The HR and SpO_2_ ring module, the O2Ring (O2Ring Smart blood oxygen ring, Lepu Medical, Shenzhen, China), is uniformly worn on the left thumb. The O2Ring includes a heart rate meter and an oximeter, which are important feedback devices for collecting physiological data. Heart rate measurements are achieved through the technique of photoplethysmography (PPG). This process capitalizes on the varying light absorption rates of vascular tissues during blood flow pulsation. The light reflected back through the skin is captured by a photo-sensitive sensor, which then translates this light into electrical signals that are subsequently digitized. From these signals, the heart rate can be deduced based on the blood’s absorbance. The computation of blood oxygen levels is based on the distinct optical properties of oxygenated hemoglobin within the human body and its differential absorption rates in response to red and infrared light. Utilizing red light at a wavelength of 660nm and near-infrared light at 940nm as the illuminating sources, the blood’s oxygen concentration and saturation levels are determined by measuring the light transmission intensity through human tissues. By monitoring the body signals in real time and fusing them with gait parameters as the input parameters of the control system, the system operates as a closed-loop system.

**Figure 2:**
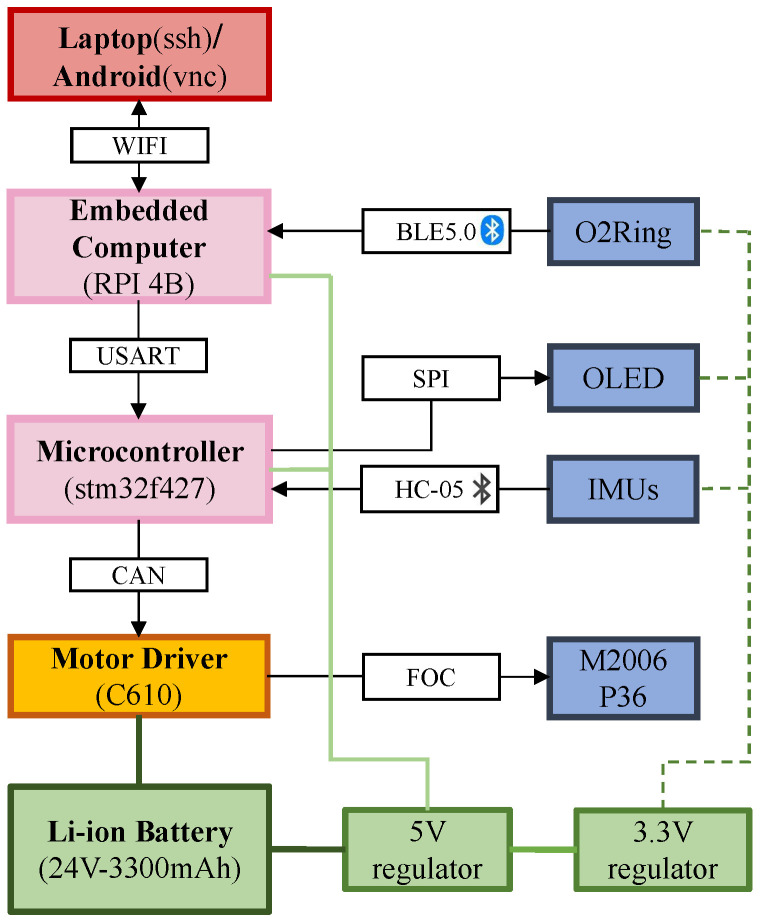
Conceptual diagram of the electronic architecture, including the processor, sensor, power components, and communication mode.

As shown in Figure 2, the control unit of the exosuit consists of three electronic modules: an advanced processing system, the Raspberry Pi 4B, which handles wireless Bluetooth functions to receive data from the O2Ring; an STMicroelectronics 32-bit Series Microcontroller Chip (STM32) control board, which supervises the low-level controller by reading motion signals from the IMU; and an electromechanical modulation C610 (Robo Master Brushless Motor Governor, DJI Innovation, Shenzhen, China), which utilizes a 32-bit custom motor driver chip and Field-Oriented Control (FOC) technology to achieve precise control of the motor torque. This is combined with a brushless DC gear motor (M2006 P36, DJI Innovation, Shenzhen, China) to form a powerful power package that manages communication through the CAN bus. The motor’s supplementary force is transferred to the hip joint through the strategic winding of a pair of synthetic tendons, made from Bowden wire fibers, in both clockwise and counterclockwise directions around the pulleys. These tendons are anchored at the upper and lower ends of each leg, ensuring a secure connection. A 24 V lithium battery (3300 mAh) powers all electronics.

### 2.2. Data Acquisition System

Junwon et al. [21] recommended an online gait task detection system for hip exoskeletons, demonstrating that the angle of the two hip joints at foot contact can accurately identify gait tasks. Zhang [22] developed a motion pattern identification and gait phase prediction model with both offline and real-time components. Baichun Wei et al. [23] used motion pattern categorization as prior knowledge to predict joint kinematics and gait events utilizing a CNN-LSTM network. However, this approach requires complex computations that can be challenging for processors to handle. In this study, IMUs were mounted on the outer ring of the thigh to detect its angular velocity in the sagittal plane. The gait cycle is initiated at the maximum angle of hip flexion, and the gait period is defined as the time between two consecutive maximal events. The initial and final trigger points are determined by the difference in thigh angles, and the value of the gait cycle time is then computed by setting an interrupt.

To verify the reasonableness and dynamics of heart rate and blood oxygen changes in different exercise states, we invited three healthy adult male subjects (178.5 ± 3.5cm, 70 ± 5kg, 25 ± 2 years old) to participate in physiological state data verification. The experiments were conducted with both fixed exercise intensity and variable exercise intensity, without wearing an exosuit. The specific physical conditions of each subject in each experiment are presented in Table 2. Data collection involved a Raspberry Pi 4B wirelessly connected to the O2Ring via Bluetooth 5.0. Another part of the data was collected by pairing the Bluetooth module of our control board with various IMUs. The sensors worn on the body were connected wirelessly and minimally. Finally, the Raspberry Pi and control board were connected via USB to a serial port for unified data storage on the Raspberry Pi. The entire physical state test consisted of two experiments. The fixed-speed experiment lasted 15 min, including 9 min of walking on a treadmill at 3.5 km/h and 3 min of standing rest before and after the trial. The variable-speed experiment involved walking on the ground for 3 min at 2km/h, then increasing the speed to 4km/h without rest for 3 min, and finally, walking at 6km/h to conclude the experiment. The total duration was 15 min, with 3 min of standing rest before and after the trial. To ensure physical recovery and allow sufficient recovery time for the subjects, each test was followed by a 10-minute rest period.

**Figure 3:**
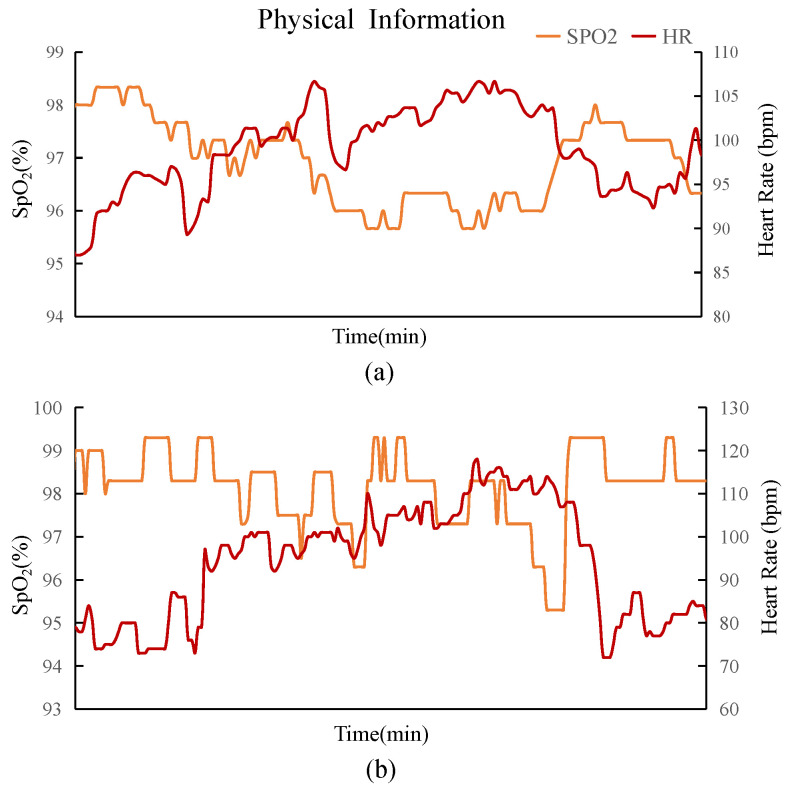
Physiological state test without a soft exosuit, showing physiological information that reflects changes in HR and SpO_2_. The fluctuation of the raw data signal may be related to motion artifacts caused by the offset of the wearer’s position during walking. (**a**) Physical changes in one of the subjects (E) walking on a treadmill at a relatively natural fixed speed (3.5 km/h). (**b**) Changes in physical signs of the same subject walking at variable speeds of 2, 4, and 6 km/h on a treadmill.

The raw data results of the physical state test are shown in Figure 3. At a normal walking speed, the changes in HR and SpO_2_ are relatively smooth, with HR increasing significantly when SpO_2_ decreases. In the case of variable speed, the physiological state changes little from static to slow speed. However, when the speed increases rapidly, HR rises significantly, and SpO_2_ decreases to a certain extent. At speeds up to 6km/h, HR is higher and SpO_2_ is lower until they return to normal levels during the stationary phase. The experiment reveals the fluctuation range and patterns of human physiological signals in different motion states, which provides a basis for the subsequent realization of a real-time control method for exosuits. Our goal is to provide the wearer with real-time, suitable support while walking; therefore, high-precision tracking is not essential for operating the soft exosuit.

## 3. Different Assistance Strategies in Different Status Conditions

### 3.1. Hip Power-Assist Strategy

**Figure 4:**
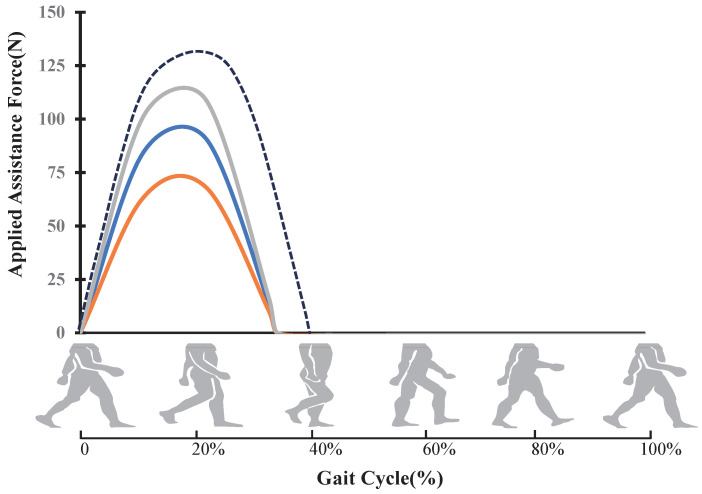
Human gait cycle analysis and examples of feasible hip force profiles.

The main phases in a whole gait cycle and examples of potential feasible hip force profiles are shown in Figure 4. Individual movement preferences are not static but should allow for a comfortable and unrestricted walking process [24]. People generally prefer movement methods that minimize energy expenditure, such as selecting appropriate speeds and step frequencies to reduce energy consumption per unit distance. Although these preferences have been established over evolutionary time, the ability to optimize energy expenditure in real time remains an unresolved issue [25]. Long-term EX1 exercise significantly improves physical function, gait, and cardiopulmonary metabolic efficiency in the elderly, particularly regarding dynamics and gait characteristics, effectively alleviating age-related physical functional decline [4]. Therefore, gait management assistive devices play an important role in treating patients with gait disorders, enhancing walking efficiency and speed, and allowing for longer and more distant walking training [26].

The soft exosuit control approach involves altering the force amplitude and phase. Different walking intensities affect joint torque, muscular activity, and ground reaction forces [27]. Changes in walking intensity affect the duration of the gait cycle, with fast walking shortening the gait cycle and slow walking extending it [28]. Fast walking or running generates greater torque and impact force than slow walking, and physiological indicators such as heart rate and oxygen saturation also increase with walking intensity [29,30]. Based on the theory of biological hip joint torque, it is necessary to design corresponding assistive torques for different exercise intensities. Traditional torque design methods rely on torque curves obtained from predetermined tests, which are inconvenient to measure in multi-state and multi-variable environments. To prevent excessive assistive torque from causing harm to subjects, this paper uses a sine curve to approximate hip joint torque [31]. By adjusting the parameters of the sine curve rather than directly changing hip joint torque, an assistive force trajectory generation algorithm based on the fusion of multiple parameters, such as the gait cycle, HR, and SpO_2_, is designed.

Equation (1) is the output expression of applying the assistive force curve at the present moment:

(1) $$F=f_{d}\;*\;\left(n+sin\left(phase*\pi /a-\pi /b\right)/2\right)*c,$$

where *F* is the force actually produced by the motor, while $f_{d}$ denotes the target force, which is determined by the algorithm based on the discrepancy in the hip joint angle. The phase is the walking phase at the present moment, and *n*, *a*, and *b* are dynamic parameters set by different users with diverse body parameters. The power factor *c* is obtained by setting weights to fuse different modes or user preferences according to physiological states.

### 3.2. Fusion Control Strategy

This study introduces an innovative control strategy for soft exosuits that not only alters the amplitude and phase of the force but also transcends traditional methods reliant on lower-limb IMU angle information. We have developed a controller that operates based on gait parameters, heart rate, and oxygen saturation data, analyzing the lower limb’s structure to obtain gait information and combining it with physiological parameters such as HR and SpO_2_ to achieve a personalized assistance scheme. These personalized options are integrated into each gait cycle and serve as a feedback channel for real-time online control. The soft exosuit is designed to minimize interference with the wearer’s normal movement and heart rate, relying on the hip joint angle for timely assistance.

The control architecture integrates both feedback and feedforward mechanisms. The feedback controller dynamically adjusts assistance based on real-time physiological parameters, while the feedforward controller anticipates movement changes by adjusting the assistance output in advance using a predictive model based on gait parameters. The system consists of a position inner loop and a proportional–derivative (PD) outer loop. The position inner loop provides positional feedback for the motor system, while the PD outer loop serves as the main controller, processing feedback data from heart rate, oxygen saturation, and gait calculations to maintain system stability [19]. The PD controller acts as an admittance feedback controller, and its operation is delineated by Equation (2):

(2) $$P_{t}=K_{p}e_{F}+K_{d}\Delta e_{F},$$

where $P_{t}$ denotes the target position of the motor; $K_{p}$ and $K_{d}$ denote the proportional and derivative controllers, respectively; and $e_{F}$ and Δ$e_{F}$ denote the errors of the current force error and its differential, respectively.

**Figure 5:**
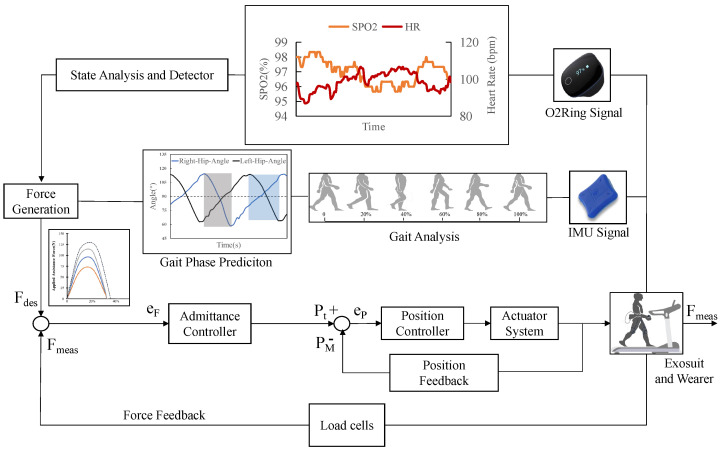
The control framework incorporates a PSMC feedback mechanism alongside an angle feedforward strategy, both operating in conjunction with a PD admittance controller.

The soft exosuit control architecture is depicted in Figure 5. Upon commencement of the robotic system, it initially acquires the angle of the hip joint through the IMU, analyzes human motion to extract gait data, and subsequently determines the walking phase. Concurrently, the system continuously monitors and calculates the gait parameters while collecting the user’s heart rate and oxygen saturation levels. By employing signal preprocessing techniques, such as filtering and noise reduction, the system ensures the accuracy and reliability of the collected data. Within each assistance cycle, the controller calculates the desired assistive torque ($F_{des}$) based on the current heart rate (HR), oxygen saturation (SpO_2_), and gait parameters. The target motor position ($P_{t}$) is determined by a combination of feedback-controlled positional signals ($P_{M}$), heart rate, and oxygen saturation feedback control signals. To aid the user in lowering metabolic expenditure, the system generates the required assistive force using the power-assist trajectory-generating approach presented in the previous section. As the user’s physiological status or gait characteristics change, such as changes in HR and gait cycle, the assistive torque at the hip joint adjusts accordingly.

## 4. Experimentation

### 4.1. Methods for Evaluating the Effectiveness of Human–Computer Interaction

Human–computer interaction needs to perceive both environmental information and human physiological information. Assessing the metabolic expenditure of exoskeleton-augmented locomotion has been established as the standard for determining the wearer’s effort and exertion [32,33]. As a result, obtaining a lower metabolic expenditure compared to unaided walking has been a primary goal for exoskeletons designed to improve strength and performance [33]. However, current wearable robotic devices frequently rely on a single or limited set of sensory modalities, which can lead to unsatisfactory performance. Numerous investigations have examined the metabolic expenditure associated with human exoskeleton-assisted ambulation [34,35]. The findings indicate that the metabolic cost during walking can be reduced with the application of suitable exoskeleton assistance modalities. The metabolic rate is frequently utilized as a physiological measure to assess the efficacy of diverse exoskeleton assistance strategies, making it a common target for human-in-the-loop (HIL) optimization [6,36]. However, the metabolic costs, which are typically assessed using indirect calorimetry, are often characterized by high noise, latency, and sparse data sampling. These attributes can result in prolonged evaluation durations, consequently hindering the efficiency of the optimization procedure [37]. Exoskeleton studies have demonstrated enhanced performance indicators, including EMG, spatio-temporal metrics, and metabolic expenditure, showing a superior range of motion and stability in various populations. Although certain parameters have been optimized for steady-state walking, performance cannot be assured when walking circumstances and behaviors change. Optimizing numerous parameters for co-adaptive and time-varying circulatory systems may require online training and optimization solutions [1,38]. Although challenging, it is possible to achieve this without costly metabolic instruments for measurement. Identifying and integrating low-cost and reliable proxies for metabolic expenditure, such as device auxiliary power or heart rate, is crucial for measuring performance [39].

### 4.2. Metabolic Consumption Experiment

#### 4.2.1. Experimental Setup of the Treadmill Walking Tests

**Figure 6:**
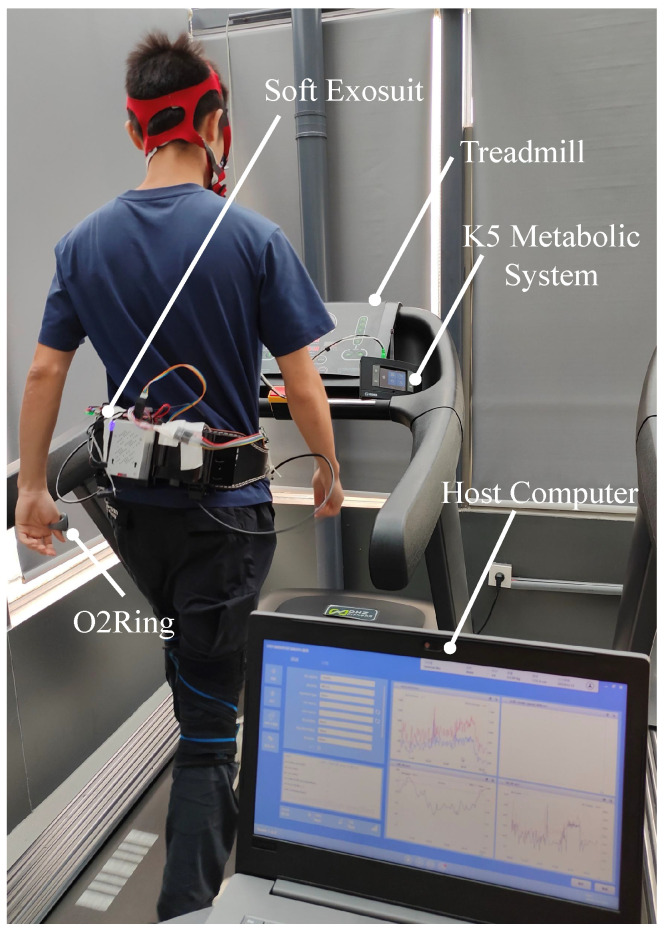
The metabolic evaluation experiment of the soft exosuit with the K5 System. A subject wears the novel soft exosuit when walking on a treadmill.

Eight healthy adult males, aged 25 ± 3 years, with a body weight of 66.8 ± 14.8 kg and a height of 174 ± 8.5 cm, were involved in the metabolic rate experiment. The experiment was conducted on a treadmill with speeds set at 2, 4, and 6 km/h, and the ambient temperature was maintained at 26 °C. To assess metabolic expenditure, we utilized a COSMED K5 respiratory gas analyzer from Rome, Italy, for quantifying exhaled gases—predominantly carbon dioxide and oxygen. Additionally, metabolic rates were determined using the modified Brockway formula [40], as articulated in the following equation:

(3) $$\Delta E=\left(c_{1}\;*\;VO_{2}+c_{2}*VCO_{2}\right)/\left(60*W\right)$$

where ΔE is the energy consumption rate measured in kilojoules per second (kJ/s) and the coefficients $c_{1}$ and $c_{2}$ are 16.89 and 4.84. The volumes of oxygen uptake and carbon dioxide emission, denoted as $VO_{2}$ and $VCO_{2}$, respectively, were ascertained using the K5 Metabolic System Figure 6. To account for variations in body mass, metabolic rates were normalized based on individual body weight. *W* denotes the weight of each subject. The basic physiological states of eight participants are shown in Table 2.

Measurements of carbon dioxide and oxygen levels were collected during the stable walking period. To minimize disruptions to the metabolic rate measurements, the experimental protocol was divided into two segments: a training phase and a testing phase. The primary goal of the orientation period was to ensure participants were adept at utilizing the various features of the soft exosuit. This was achieved through a key exercise that required participants to operate the exosuit in both standard and advanced modes: one without an integrated strategy and another with an integrated strategy for assistance. Observations revealed that, under the integrated assistance mode, there was no pronounced fluctuation or irritation in the heart rate and blood oxygen levels of the wearers until they became accustomed to wearing the soft exosuit. Subsequently, participants were instructed to walk on a treadmill at a fixed speed and at three variable speeds for a minimum duration of 5 min per speed.

#### 4.2.2. Metabolic Reduction by the Exosuit in Fixed-Speed Conditions

**Figure 7:**
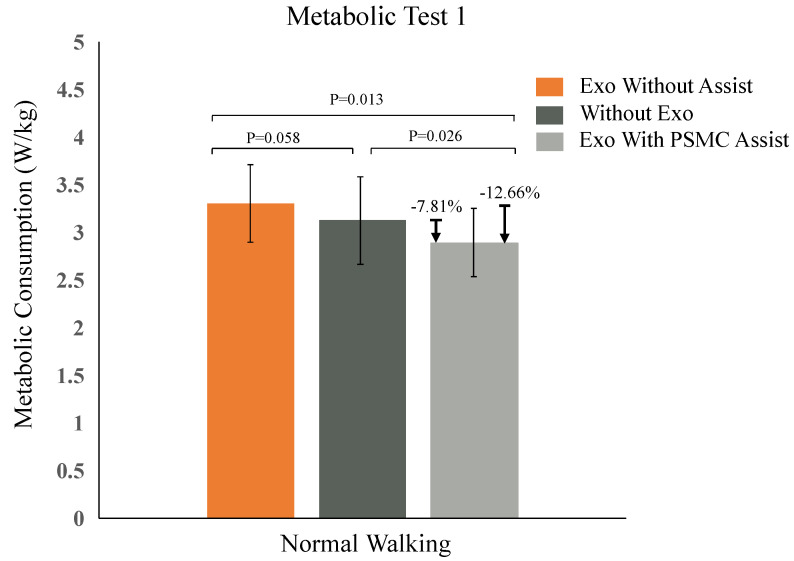
The metabolic assessment was conducted across three scenarios: exosuit used without aid, non-used of an exosuit, and exosuit used with aid. The ‘*p*’ values, derived from two-sided *t*-tests, were 0.058, 0.026, and 0.013 for each condition respectively.

During the initial phase, participants underwent three distinct metabolic assessments: one without the exosuit, one with the unassisted exosuit, and one with the exosuit providing support during treadmill walking (with the treadmill speed set at 3.5 km/h). Each trial lasted for 15 min, with the initial 5 min dedicated to measuring the resting metabolic rate in a stationary posture. Afterward, participants resumed a standing position to rest and allow for the observation of heart rate and blood oxygen signals. The net metabolic rate was determined by calculating the difference between the exercise metabolic rate and the resting metabolic rate, with the group assisted by the soft exosuit serving as a reference baseline. By comparing this with the metabolic rates of the non-wearing group and the wearing-but-unassisted group, the value of metabolic reduction was determined, respectively. As depicted in Figure 7, it was demonstrated that the net metabolic rate decreased significantly by 7.81% and 12.66% when comparing walking with the soft exosuit (PSMC mechanism) to walking without it and walking with the unpowered exosuit use, respectively.

#### 4.2.3. Metabolic Reduction by the Exosuit in Variable-Speed Conditions

**Figure 8:**
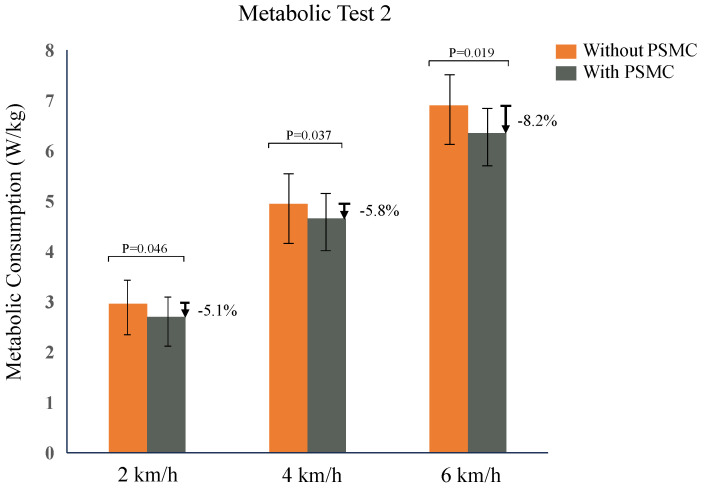
Metabolic costs vary with and without the PSMC method in exosuit-assisted subjects.

During the variable-speed testing phase, participants initially walked on level ground both with and without the soft exosuit, followed by two experimental sets while wearing the exosuit: one utilizing the PSMC strategy and the other without it. Given the significant impact of muscle fatigue on metabolic expenditure, a half-hour interval was allocated between each trial to allow participants to recuperate from muscular fatigue. On the days designated for experimental testing, the sequence of trials was randomized for each individual. Each trial was set to last 25 min, with the initial 5 min dedicated to measuring the resting metabolic rate in a stationary posture. Subsequently, participants were requested to walk for a period of 5 min to ascertain the metabolic rate during exercise for each speed. The other steps were the same as in the previous experiment. The net metabolic rate of the group receiving assistance from the soft exosuit with the PSMC method was established as the reference point. The metabolic reduction was ascertained by comparing it against the metabolic rates of the group without the PSMC method. Figure 8 displays the subjects’ metabolic rates from each experiment. The results support the assumption that the mode with the fastest speed has the highest metabolic rate. The soft exosuit reduced the net metabolic rate by 5.1%, 5.8%, and 8.2% for each speed, with absolute decreases of 0.26 W/kg, 0.29 W/kg, and 0.55 W/kg.

## 5. Discussion

This research introduces the innovative design of a soft exosuit, specifically tailored for hip joint assistance, and seamlessly incorporates inputs derived from human physiology. A fusion control method based on heart rate, blood oxygen, and gait parameters is designed to ensure the accuracy and personalization of assistance. An evaluation of the performance of the proposed control strategy has been conducted, including a comparative analysis of the net metabolic cost across various assistance modalities. The exoskeleton system is lightweight and portable. The weight of this prototype is slightly lighter than that of existing powered portable hip-assisted soft external protectors. This study presents the design of a novel lower-limb joint soft exoskeleton that provides hip flexion assistance. The auxiliary profile is established for motion intensities at three different speeds, and the controller is designed based on a strategy that fuses physiological signals with a feedforward model. The effectiveness of the control strategy, as well as the overall metabolic expenditure associated with varying exercise intensities at multiple speeds, has been comprehensively assessed.

The reduction in net metabolic expenditure is associated with the support provided to the lower limbs, particularly the hip joint. The assistance setting is closely related to the performance of human–computer interaction. The perception of the physiological state during human movement greatly contributes to the realization of human-in-the-loop systems. An intuitive idea is that fusing physiological state data with gait preference parameters using this method will greatly reduce the metabolic rate.

Although the study in [15] noted that the booster settings could be adjusted based on user preferences, it did not go into detail about how to personalize the booster mode based on individual physiological parameters. Using individualized interaction, we designed a soft exosuit that assists the hip joint to minimize the metabolic cost of walking under different physiological states and exercise intensities. The final results demonstrate the feasibility of our hip assistance scheme. A decline in metabolic expenditure was observed across three varying levels of exercise intensity, with the most significant decrease occurring at the highest walking velocity. Walking at the highest speed reduced the maximal metabolic rate by 7.81% compared to walking without the soft exosuit. Given that the torques in the joints of the lower limbs fluctuate depending on individual gait patterns and the intensity of the exercise, it can be inferred that identical assistive approaches may yield varying impacts on metabolic expenditure. Investigations [22] and [41] explored the impact of gait phase detection and the application of auxiliary force curves on reducing metabolic expenditure. Based on the biological torque of the hip joint, three auxiliary strategies were established for the three velocity conditions formed by motion intensities of 2, 4, and 6 km/h. The effects of these assistive strategies varied for different speeds and body states. However, a specific relationship between aid strategy, speed, and exercise intensity was not determined. In subsequent research endeavors, we aim to establish additional motion states, thereby enhancing our capacity to devise more effective support strategies. Beyond considering biological torques, employing a HIL methodology also serves as a viable alternative for ascertaining suitable assistive measures, as demonstrated in [42,43] for optimizing assistive forces to improve performance and reduce metabolic rates.

## 6. Conclusions

A key objective of designing a soft exosuit is to alleviate the user’s energy expenditure by offering support while simultaneously minimizing the metabolic burden imposed by the system’s weight. Walking at different exercise intensities leads to varying biomechanics, and at the same time, the physiological state changes. However, researchers have rarely studied the assistive forces across different motion states. In our research, we introduce an innovative support strategy, known as Physiological State Monitoring Control (PSMC), aimed at enhancing the functionality of an exosuit system for aiding hip flexion. Drawing from the biomechanical principles governing human locomotion, we have formulated three distinct supportive strategies. Furthermore, we have integrated a composite control approach to enhance the system’s capacity for real-time responsiveness and the quality of human–machine interaction. The feedback loop in the controller allows the user to adjust the power-assist level during movement. Discrepancies between the wearer’s biometrics and the exosuit’s positioning are minimized, thereby mitigating associated errors, as corroborated by the outcomes of the tracking experiment. A comprehensive suite of experiments was conducted to substantiate the efficacy of our innovative approach in delivering auxiliary torques across varying physiological conditions. The results indicate decreases in the net metabolic rate of 5.1%, 5.8%, and 8.2% at walking velocities of 2 km/h, 4 km/h, and 6 km/h, respectively, when comparing the soft exosuit employing the PSMC method to when the exosuit was utilized without the PSMC method.

This research demonstrates that a personalized approach to hip exosuit robotics, leveraging integrated parameters, significantly contributes to the reduction of walking’s metabolic expenditure. In addition, changes in heart rate and blood oxygen parameters can reflect the assistance provided by the exosuit to the human body’s state, offering a simple and feasible evaluation method compared to the more tedious oxygen consumption tests for evaluating exosuit performance. Due to the light weight of the exosuit, its auxiliary performance and adaptability to different exercise intensities achieved satisfactory results, making it suitable for assisting healthy people, especially the elderly. Future research will explore the effects of aid on complex terrains and develop corresponding aid strategies.

## Figures and Tables

**Table 1:** The mass distribution of the novel soft exosuit.

Part	Location	Mass (kg)
Batteries	Waist	0.55
Actuator	Waist	0.214
Raspberry pi	Waist	0.106
MCU	Waist	0.08
Waist belt	Waist	0.30
IMU	Thigh	0.024
Load cells	Thigh	0.05
Wraps	Thigh	0.22
O2Ring ring	Finger	0.015

**Table 2:** The physiological states of the participants.

Subjects	Age (Years)	Gender	Height (cm)	Weight (kg)	Physiological State Test (Y/N)
A	24	Male	170	75	
B	25	Male	173	66	
C	23	Male	175	70	Y
D	25	Male	172	52	
E	25	Male	176	65	Y
F	26	Male	177	74	
G	26	Male	165	58	
H	27	Male	182	75	Y

## Data Availability

The readers can obtain data from the authors.

## Abbreviations

The following abbreviations are used in this manuscript:

HR	Heart rate
SpO_2_	Blood oxygen saturation
IMU	Inertial Measurement Unit
STM32	STMicroelectronics 32-bit Series Microcontroller Chip
PSMC	Physiological State Monitoring Control
EMG	Electromyography
PD	Proportional-derivative %TLA & Three-letter acronym % RMS & Root Mean Square
